# Analysis of different model-based approaches for estimating dFRC for real-time application

**DOI:** 10.1186/1475-925X-12-9

**Published:** 2013-01-31

**Authors:** Erwin J van Drunen, J Geoffrey Chase, Yeong Shiong Chiew, Geoffrey M Shaw, Thomas Desaive

**Affiliations:** 1 University of Canterbury, Christchurch, 8041, New Zealand; 2 Christchurch Hospital, Christchurch, 8011, New Zealand; 3 University of Liège, Liège, Belgium

**Keywords:** Mechanical ventilation, Functional residual capacity, FRC, dFRC, PEEP, Pulmonary, Model-based methods, ARDS, Intensive care, ICU

## Abstract

**Background:**

Acute Respiratory Distress Syndrome (ARDS) is characterized by inflammation, filling of the lung with fluid and the collapse of lung units. Mechanical ventilation (MV) is used to treat ARDS using positive end expiratory pressure (PEEP) to recruit and retain lung units, thus increasing pulmonary volume and dynamic functional residual capacity (dFRC) at the end of expiration. However, simple, non-invasive methods to estimate dFRC do not exist.

**Methods:**

Four model-based methods for estimating dFRC are compared based on their performance on two separate clinical data cohorts. The methods are derived from either stress-strain theory or a single compartment lung model, and use commonly controlled or measured parameters (lung compliance, plateau airway pressure, pressure-volume (PV) data). Population constants are determined for the stress-strain approach, which is implemented using data at both single and multiple PEEP levels. Estimated values are compared to clinically measured values to assess the reliability of each method for each cohort individually and combined.

**Results:**

The stress-strain multiple breath (at multiple PEEP levels) method produced an overall correlation coefficient R^2^ = 0.966. The stress-strain single breath method produced R^2^ = 0.530. The single compartment single breath method produced R^2^ = 0.415. A combined method at single and multiple PEEP levels produced R^2^ = 0.963.

**Conclusions:**

The results suggest that model-based, single breath and non-invasive approaches to estimating dFRC may be viable in a clinical scenario, ensuring no interruption to MV. The models provide a means of estimating dFRC at any PEEP level. However, model limitations and large estimation errors limit the use of the methods at very low PEEP.

## Background

Patients suffering from severe respiratory insufficiency such as Acute Respiratory Distress Syndrome (ARDS) (mild, moderate, severe) [1] are admitted to the intensive care unit (ICU) and require mechanical ventilation (MV) for breathing support. ARDS is associated with lung inflammation and fluid filling causing a loss of functional lung units resulting in a stiffer lung with reduced intrapulmonary gas volume known as the “baby lung” [2]. Mortality rates for ARDS have been reported to be between 20% to 70% [3].

Clinicians offer a supportive environment that aids ARDS patient recovery by application of MV. However, further harm can result from suboptimal MV [4]. Typically, the severity of ARDS is measured as the ratio of the arterial partial pressure of oxygen divided by the fraction of inspired oxygen (PaO_2_/FiO_2_ ratio). A PaO_2_/FiO_2_ value less than 300mmHg implies the patient has mild ARDS, while less than 200mmHg is moderate ARDS and less than 100mmHg is characterised as severe ARDS [1].

Functional residual capacity (FRC) represents the pulmonary gas volume of the lung at zero end expiratory pressure (ZEEP), i.e. at atmospheric pressure after normal expiration. Positive end expiratory pressure (PEEP) is applied to ARDS patients to maintain recruitment during subsequent breathing cycles [5-8]. PEEP improves gas exchange and ensures pulmonary volume above FRC. However, there is a risk of overstretching healthy lung units during high PEEP [9]. The optimal PEEP remains highly debated with no conclusive results [5], and setting this parameter is thus a balance between high and low values. Given the impact of MV on cost and length of stay [10], ensuring an optimal PEEP would have significant impact.

**Figure 1:**
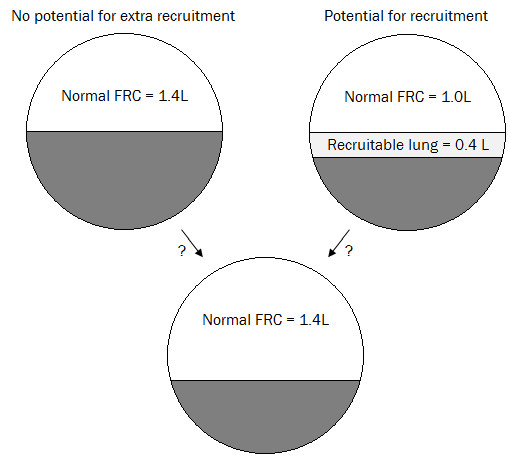
Schematic of lung showing the limitation of an absolute FRC value.

Figure 1 shows a schematic of the lung. An absolute value of FRC gives no information on the potential for new recruited lung volume during MV. A lung with an FRC of 1.4L could be a result of a lung with 1.4L of fully recruited healthy lung units or 1.0L of recruited lung plus an additional amount of lung recruited due to additional PEEP. Knowing this difference would allow PEEP to be optimized to maximize recruitment and ensure any increase in PEEP added recruited lung volume.

Currently, there are few methods of measuring FRC at the bedside. Gas washout/washin techniques are one method [11], but are not necessarily available on most ventilators. FRC can also be measured by using chest imaging methods such as Computed Tomography (CT) scans [12] and Electrical Impedance Tomography (EIT) scans [13,14]. Timed at the end of expiration, the lung volume can be assessed at each CT or EIT slice and summed across all the slices in the lung to evaluate true lung FRC. However, this type of measurement is unrealistic for regular use in guiding MV or continuous monitoring in the ICU. Although specialised ventilators can measure FRC and re-estimate FRC following PEEP changes (GE, Engstrom, Carestation ventilators), most standard ventilators cannot. Thus, there is motivation to estimate the PEEP induced FRC change to avoid further lung injury and complication.

**Figure 2:**
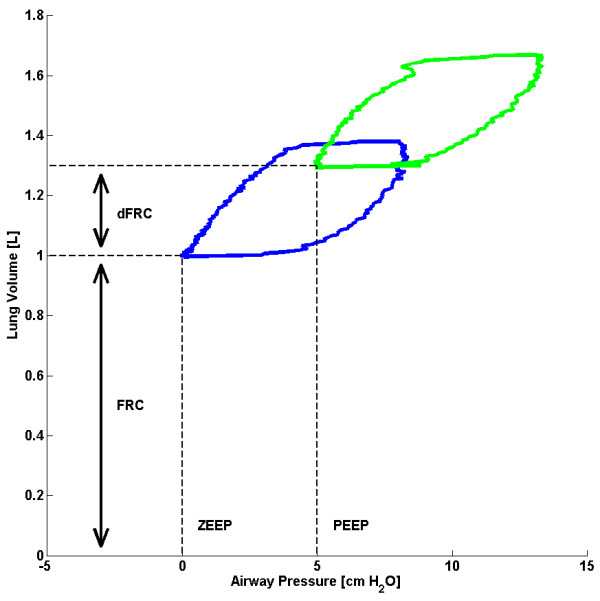
Schematic showing the difference between FRC and dFRC.

The level of additional lung volume due to additional PEEP is known as dynamic FRC (dFRC) [15] and is shown schematically in Figure 2. The ability to use standard ventilator data to simply and non-invasively estimate dFRC without interrupting MV treatment would be a significant potential enhancement in ventilation management. Although dFRC cannot by itself estimate the potential of lung recruitment, used with arterial blood gas measurements it can provide the clinician with useful information on lung recruitability as PEEP or other MV settings are modified. Thus, dFRC represents an aspect of the primary clinical endpoint in ventilation management, with the potential to be continuously tracked with changes in patient condition.

In this study, four model-based methods of estimating dFRC are presented and their performance investigated. The first method, proposed by Sundaresan et al. [15], is based on a stress–strain approach. It requires pressure-volume (PV) data at a minimum of two PEEP levels. Additional PV loops at a wide range of PEEP levels are required for higher accuracy thus requiring an interruption to ongoing MV treatment. The second method, proposed by Mishra et al. [16] is an extension to [15] and only requires PV data from a single breath at one PEEP level to estimate dFRC for a given patient eliminating interruption. The third method is based on a single compartment lung model and also requires PV data from a single breath at one PEEP level. The final method is a combination of the methods proposed by [15] and [16] with the aim of providing both accuracy and clinical functionality as data becomes available. All four methods are tested on two separate clinical data cohorts where both PV data and dFRC were measured directly.

## Methods

### Stress-strain multiple breath method (SSMB)

Chiumello et al [17] proposed a stress-strain theory of lung dynamics where the transpulmonary pressure (∆*P*_*L*_) is defined as the clinical equivalent of stress. Transpulmonary pressure is the difference between the applied airway pressure and the corresponding pleural pressure. The clinical equivalent of strain is the ratio of the change in volume (∆*V*) to the FRC, which represents the resting lung volume, yielding a stress-strain definition:

(1) $$\Delta P_{L}\left(\text{stress}\right)=E_{L,\mathrm{spec}}\times \frac{\mathrm{\Delta V}}{\text{FRC}}\left(\mathrm{strain}\right)$$

where the specific lung elastance (*E*_*L*,*spec*_) can be defined as the transpulmonary pressure at which FRC effectively doubles. The general relationship between the plateau airway pressure (∆*P*_*aw*_), when the airflow is zero, and the corresponding transpulmonary pressure is defined as [17]:

(2) $$\Delta P_{L}\left(\text{stress}\right)=\Delta P_{\text{aw}}\times \alpha$$

(3) $$\alpha =\frac{E_{L}}{E_{L}+E_{\text{CW}}}$$

where *α* represents the static lung elastance and represents the ratio of the lung elastance (*E*_*L*_) to the chest wall elastance (*E*_*CW*_). The value of *α* indicates the severity of ARDS, where a higher value of *α* indicates a higher severity of ARDS [15,18]. The equation of motion describing the airway pressure as a function of the resistive and elastic components of the respiratory system is defined as [19]:

(4) $$\Delta P_{\text{aw}}=V\times E_{\text{rs}}+Q\times R_{\text{rs}}+P_{o}$$

where *V* is the volume ranging from zero at the beginning of inspiration to the tidal volume (*V*_*t*_) at the end of inspiration, *E*_*rs*_ is the respiratory elastance, *Q* is the airflow rate, *R*_*rs*_ is the respiratory resistance and *P*_*o*_ is the offset pressure, which is a combination of applied PEEP and intrinsic PEEP [20,21]. All of the PEEP data in this study represent plateau airway pressures, which are measured at zero airflow at end expiration. Because transpulmonary pressure is not typically measured at the bedside, it is estimated using the PEEP. Thus, rather than using the transpulmonary pressure, the airway pressure is used as an estimate based on Eq. (2). If the pressure and volume are measured at the point between expiration and inspiration, where the flow is zero, the resistive term is zero and the airway pressure is equal to the plateau airway pressure. Combining Eqs. (1) and (2) yields a formula for FRC involving ∆*V*=∆*dFRC* and ∆P_*aw*_=∆*PEEP*:

(5) $$\text{FRC}=\frac{\text{ΔdFRC}}{\text{ΔPEEP}}\times \frac{E_{L,\text{spec}}}{\alpha }$$

Eq. (5) defines FRC as a function of the volume responsiveness of the patient to the specified change in PEEP, $\frac{\text{ΔdFRC}}{\text{ΔPEEP}},E_{L,\mathrm{spec}}$ and *α* of the patient. In this model, FRC and the effect on FRC from a recruitment manoeuvre are not known. Thus, it was hypothesised that dFRC follows a similar mathematical form to Eq. (5):

(6) $$\text{FRC}+\text{dFRC}=\frac{\text{ΔdFRC}}{\text{ΔPEEP}}\times \frac{E_{L,\mathrm{spec}}}{\alpha }\left(1+x\right)$$

Therefore, dFRC takes the form:

(7) $$\text{dFRC}=\frac{\text{ΔdFRC}}{\text{ΔPEEP}}\times \frac{E_{L,\text{spec}}}{\alpha }x$$

where *x* is a function of the PEEP level at which dFRC is estimated. *E*_*L*,*spec*_ and *α* are relatively constant parameters [17] so can be combined into one unknown parameter, *β* yielding:

(8) $$\text{dFRC}=\frac{\text{ΔdFRC}}{\text{ΔPEEP}}\times \beta$$

where *β* is a function of the PEEP, *E*_*L*,*spec*_ and *α*. The assumption that *α* is constant is true only for the linear portion of the static PV curve [15]. The value of *β* for a single value of PEEP is assumed constant across all patients. An additional file shows the *β* values determined for each data cohort [see Additional file 1. Because FRC was not known for any patient, *β* was analytically solved based on Eq. (8) using measured dFRC values from the data. Once *β* values were evaluated for each patient at each PEEP level, a median *β* was then evaluated at each PEEP level to serve as a population constant for that PEEP level. The dFRC was then estimated using Eq. (8) and the median *β* value. The process can be summarised as follows:

1. Analytically solve Eq. (8) to find *β* for each patient and PEEP.

2. Evaluate population based median *β* at each PEEP level.

3. Estimate dFRC using Eq. (8) and the population based median *β*.

This method requires the patient to undergo a stepwise PEEP increase manoeuvre to obtain multiple PV loops at different PEEP levels prior to analysis.

### Stress-strain single breath method (SSSB)

Mishra et al. [16] proposed a model to estimate ∆dFRC using only data from a single PEEP level. Once again, combining Eqs. (1) and (2) yields a formula for FRC involving ∆*V*=*V*_*t*_ and ∆*P*_*aw*_:

(9) $$\text{FRC}=\frac{V_{t}}{\Delta P_{\text{aw}}}\times \frac{E_{L,\mathrm{spec}}}{\alpha }$$

Eq. (9) defines FRC as a function of the volume responsiveness of the patient to the specified change in airway pressure observed during inspiration, $\frac{V_{t}}{\Delta P_{\text{aw}}},E_{L,\text{spec}}$ and *α* of the patient. In this model, FRC and the effect on FRC from a recruitment manoeuvre are not known. Thus it was hypothesised that ΔdFRC follows a similar mathematical form to Eq. (9):

(10) $$\text{FRC}+\text{ΔdFRC}=\frac{V_{t}}{\Delta P_{\text{aw}}}\times \frac{E_{\text{Lspec}}}{\alpha }\left(1+x\right)$$

Therefore, ΔdFRC takes the form:

(11) $$\text{ΔdFRC}=\frac{V_{t}}{\Delta P_{\text{aw}}}\times \frac{E_{\text{Lspec}}}{\alpha }x$$

Once again, combining *x*, *E*_*L*,*spec*_ and *α* into one unknown parameter, *β* yields:

(12) $$\text{ΔdFRC}=\frac{V_{t}}{\Delta P_{\text{aw}}}\times \beta$$

where *β* is a function of the PEEP, *E*_*L*,*spec*_ and *α*. As with the SSMB method, the assumption that *α* is constant is true only for the linear portion of the static PV curve [15]. The value of *β* for a single value of PEEP is assumed constant across all patients. Calculated *β* values were normalized by tidal volume as dFRC can vary with the applied tidal volume [16].

(13) $$\beta_{1}=\frac{\beta }{V_{t}}$$

An additional file shows the *β*_1_ values determined for each data cohort [see Additional file 1]. Values of *β* and ∆dFRC were calculated through the same approach as outlined for the SSMB method.

### Single compartment single breath method (SCSB)

An alternative method of estimating dFRC without the use of a population constant uses the single compartment linear lung model [19] as defined by Eq. (4). Assuming *P*_o_ is also the pressure to increase baseline FRC, then *P*_o_ can be defined:

(14) $$P_{o}=\text{PEEP}=E_{\text{rs}}\times V_{\mathrm{Po}}$$

where, *V*_*Po*_ is the additional lung volume increase due to PEEP. Substituting Eq. (14) into (4) gives:

(15) $$\Delta P_{\mathrm{aw}}=\left(V+V_{\text{Po}}\right)\times E_{\text{rs}}+Q\times R_{\text{rs}}$$

Eq. (14) could be an alternative method to estimate dFRC using respiratory elastance [22]. In particular, Eq. (14) is used to calculate *V*_*Po*_ which from Eq. (15) is expected to capture the change in FRC due to PEEP changes, thus *V*_*Po*_ is proportional to dFRC. The respiratory elastance in this study was determined using the integral based method [23] for the inspiration portion of the measured breathing cycle.

### Combined method (CM)

This model-based approach is intended for real-time clinical use in the ICU. Initially, when data at only one PEEP level is available, the model relies on the SSSB analysis [16]. As additional higher PEEP settings are introduced during the course of care, the model converts from SSSB to SSMB analysis [15]. Therefore, the model can predict dFRC at any PEEP level with the potential advantage of increasing accuracy as different PEEP settings are progressively introduced. This approach presents a non-invasive method that utilizes all available and prior data. This method aims to combine the higher accuracy of the SSMB method with the higher clinical feasibility of the SSSB method.

### Clinical patient data

Retrospective clinical data was used, consisting of 10 patients (each) from Sundaresan [24] and Bersten et al. [9] (cohorts 1, 2 respectively). For cohort 1, the dFRC was calculated during post-processing of flow data obtained by a pneumotachometer. The difference in flow rate across a PEEP change was used to estimate dFRC. For cohort 2, the dFRC was calculated directly by deflation to ZEEP at the end of a breathing cycle for each PEEP level. The demographics and cause of lung injury of all patients are shown in Table 1. The PEEP levels at which data was obtained for each cohort is also presented [see Additional file 2.

**Table 1:** Characteristics of the patients

	**Patient**	**Sex**	**Age** [**years**]	**Cause of Lung Injury**
Cohort 1 [24]	1	Female	61	Peritonitis
	2	Male	22	Trauma
	3	Male	55	Aspiration
	4	Male	88	Pneumonia
	5	Male	59	Pneumonia
	6	Male	69	Trauma
	7	Male	56	Legionnaires
	8	Female	45	Aspiration
	9	Male	37	H1N1
	10	Male	56	Legionnaires
Cohort 2 [9]	1	Male	74	Ruptured AAA
	2	Male	24	Lung contusion
	3	Female	72	Legionnaires
	4	Male	48	Pancreatitis
	5	Female	68	Pulmonary embolus
	6	Male	54	Aspiration
	7	Male	73	Aspiration
	8	Male	72	Pneumonia
	9	Male	81	Aspiration
	10	Male	47	Liver transplant

### Analysis

Estimated dFRC values were compared with the clinically measured dFRC to determine the estimation error over each method and data cohort. Performance was assessed by trend correlation coefficient (R^2^) where comparisons between measured and estimated values were made. The maximum, minimum, median and interquartile range non-parametric statistics were chosen to be the summary statistics to display. The accuracy of each method was compared in relation to the other methods and the functionality of each method was evaluated.

## Results

### Stress-strain multiple breath method (SSMB)

**Figure 3:**
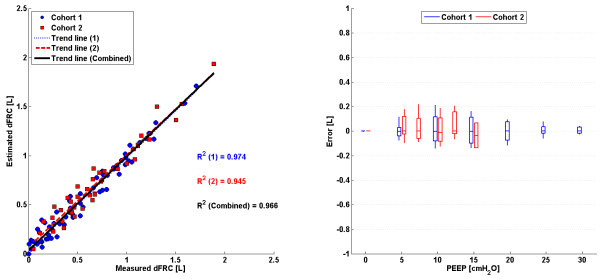
**SSMB: Plots of measured dFRC vs**. **estimated dFRC and associated error.** (Left) Plot of clinically measured dFRC vs. estimated dFRC for all patients and PEEP levels. (Right) Box plot of errors between clinically measured dFRC and estimated dFRC for all patients and PEEP levels.

The linear trend in clinical vs. predicted dFRC across all PEEP levels and the associated error for each cohort is shown in Figure 3. Values of R^2^ are given, for both cohorts 1 and 2 separately and combined.

### Stress-strain single breath method (SSSB)

**Figure 4:**
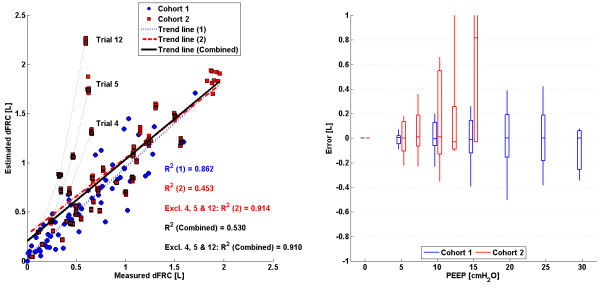
**SSSB: Plots of measured dFRC vs**. **estimated dFRC and associated error.** (Left) Plot of clinically measured dFRC vs. estimated dFRC for all patients and PEEP levels. Patient specific trends are indicated for the cases of significant overestimation. (Right) Box plot of errors between clinically measured dFRC and estimated dFRC for all patients and PEEP levels. Errors larger than ±1L are truncated for clarity.

The linear trend in clinical vs. predicted dFRC across all PEEP levels and the associated error for each cohort is shown in Figure 4. Values of R^2^ are given, for both cohorts 1 and 2 separately and combined. Values of R^2^ are also given for the cases where outlying patients have been excluded.

### Single compartment single breath method (SCSB)

**Figure 5:**
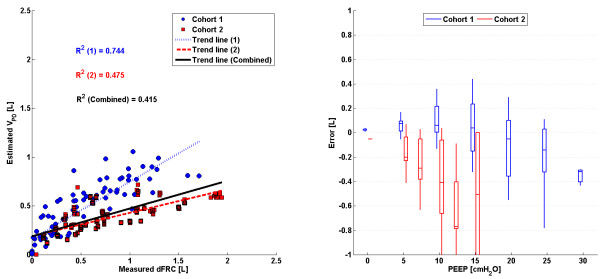
**SCSB: Plots of measured dFRC vs**. **estimated dFRC and associated error.** (Left) Plot of clinically measured dFRC vs. estimated *V*_*Po*_ for all patients and PEEP levels. (Right) Box plot of errors between clinically measured dFRC and estimated *V*_*Po*_ or all patients and PEEP levels. Errors larger than ±1L are truncated for clarity.

The linear trend in clinical dFRC vs. predicted *V*_*Po*_ across all PEEP levels and the associated error for each cohort is shown in Figure 5. Values of R^2^ are given, for both cohorts 1 and 2 separately and combined.

### Combined method (CM)

**Figure 6:**
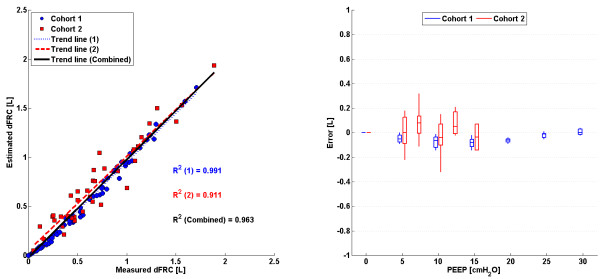
**CM: Plots of measured dFRC vs**. **estimated dFRC and associated error.** (Left) Plot of clinically measured dFRC vs. estimated dFRC for all patients and PEEP levels. (Right) Box plot of errors between clinically measured dFRC and estimated dFRC for all patients and PEEP levels.

The linear trend in clinical vs. predicted dFRC across all PEEP levels and the associated error for each cohort is shown in Figure 6. Values of R^2^ are given, for both cohorts 1 and 2 separately and combined.

Table 2 summarizes the correlation coefficients for each method and cohort.

**Table 2:** **Summary of trend correlation coefficients** (**R**^**2**^) **for each method and cohort**

	**Cohort 1**[24]	**Cohort 2**[9]	**Overall coefficient**
SSMB	0.974	0.945	**0**.**966**
SSSB	0.862	0.453	**0**.**530**
SCSB	0.744	0.475	**0**.**415**
CM	0.991	0.911	**0**.**963**

## Discussion

### Stress-strain multiple breath method (SSMB)

**Figure 7:**
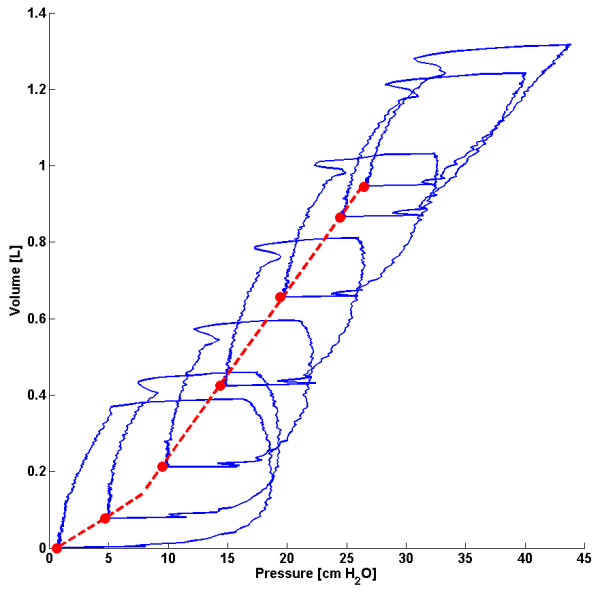
**PV loops for patient 1** (**cohort 1**) **indicating a change in the trend of compliance at an auto**-**PEEP of 7cmH**_**2**_**O**.

There exists a strong, sustained linear trend in measured vs. estimated dFRC over all PEEP values and a wide range of dFRC for each cohort. Auto-PEEP, present in some patients from cohort 1 [24], may have affected the correlation coefficient for that cohort. Auto-PEEP has the effect of a sudden change in the level of recruitment (ΔdFRC) once the PEEP becomes greater than the auto-PEEP, as shown in Figure 7 for an auto-PEEP of 7cmH_2_O. Although cohort 1 contained patients with auto-PEEP, the median error, as shown in Figure 3, was consistently small across all PEEP levels indicating no inherent tendency for overestimation or underestimation. A significant drawback with this method is that it assumes a linear compliance trend across all PEEP levels which may not hold true for cases where auto-PEEP is present, or at high PEEP levels where overdistension can occur. Another limitation with this model is that it requires PV data from at least two PEEP levels so it cannot be used for continuous tracking of dFRC. Its application in real-time dFRC measurement is thus limited without interrupting MV treatment.

### Stress-strain single breath method (SSSB)

The single breath method proposed by Mishra et al. [16] and applied to cohort 2 [9] resulted in the lowest overall correlation in the study. This result is specifically due to the trend of trials 4, 5 and 12, as shown in Figure 4, where the error reached as high as 1.66L. This large error was caused by two factors:

1. All three trials exhibited a relatively low compliance trend when compared to the majority of other trials in the cohort, resulting in a lower calculated *β*_1_ value. The population constant *β*_1_ value was calculated as the median of all *β*_1_ values at a given PEEP. Hence, a significantly higher median *β*_1_ value was, in turn, applied to these patients, causing error.

2. Several other trials also exhibited reasonably low compliance trends, but did not result in overestimation. This difference in outcome occurs because values of *β*_1_ were normalised by *V*_*t*_. Generally, trials 4, 5 and 12 had higher *V*_*t*_ when compared to other trials exhibiting the same trend in compliance.

Combined, these two factors resulted in considerable overestimation of dFRC for these three trials and potentially highlight a significant limitation with this method. No patient-specific or case-specific factor could be identified as the root cause of this difference. However, it should be noted that measurements for patients in cohort 1 were obtained with no prior recruitment manoeuvre or stabilization and had a far higher R^2^ value of 0.862 as shown in Figure 4 and Table 2. In contrast, the study of [9] recruited and stabilized patients at each PEEP level for 30 minutes. Thus, as a result, this cohort may see higher *V*_*t*_ despite low compliance, which is a scenario not typically seen clinically.

An advantage of the SSSB method over the SSMB method is that a unique value of *β*_1_ is determined at each PEEP level which is independent of lung behaviour at other PEEP levels. Thus, values of *β*_1_ can account for the natural sigmoidal nature of a patients’ volume responsiveness to PEEP increase. This is important for cases where auto-PEEP is present, or at high PEEP levels where overdistension can occur. A disadvantage is that the calculated values of *β*_1_ are dependent on the method of data measurement. As previously mentioned, patients in cohort 2 were stabilized prior to measurement while those in cohort 1 were not. Thus, the *β*_1_ values obtained from cohorts 1 and 2 diverge as PEEP increases. Combining these cohorts to obtain a larger population for determining values of median *β*_1_ would result in poorer dFRC estimation across both cohorts.

### Single compartment single breath method (SCSB)

**Figure 8:**
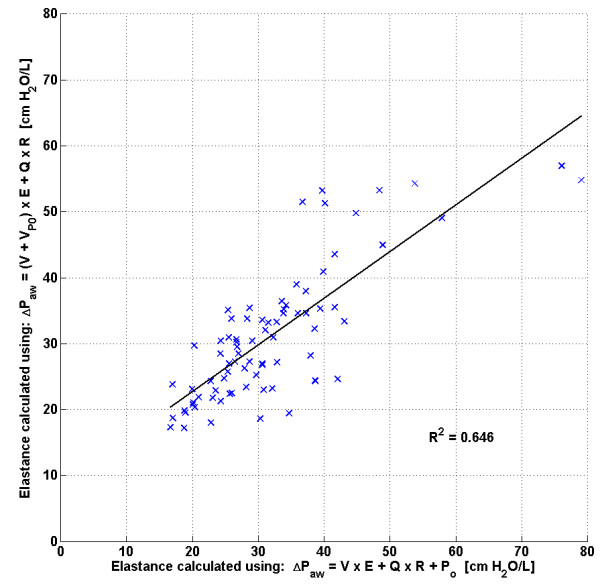
**Comparison between respiratory elastance when calculated using Eq**. (**4**) **and Eq**. (**15**) **for all patients in cohort 1**.

**Figure 9:**
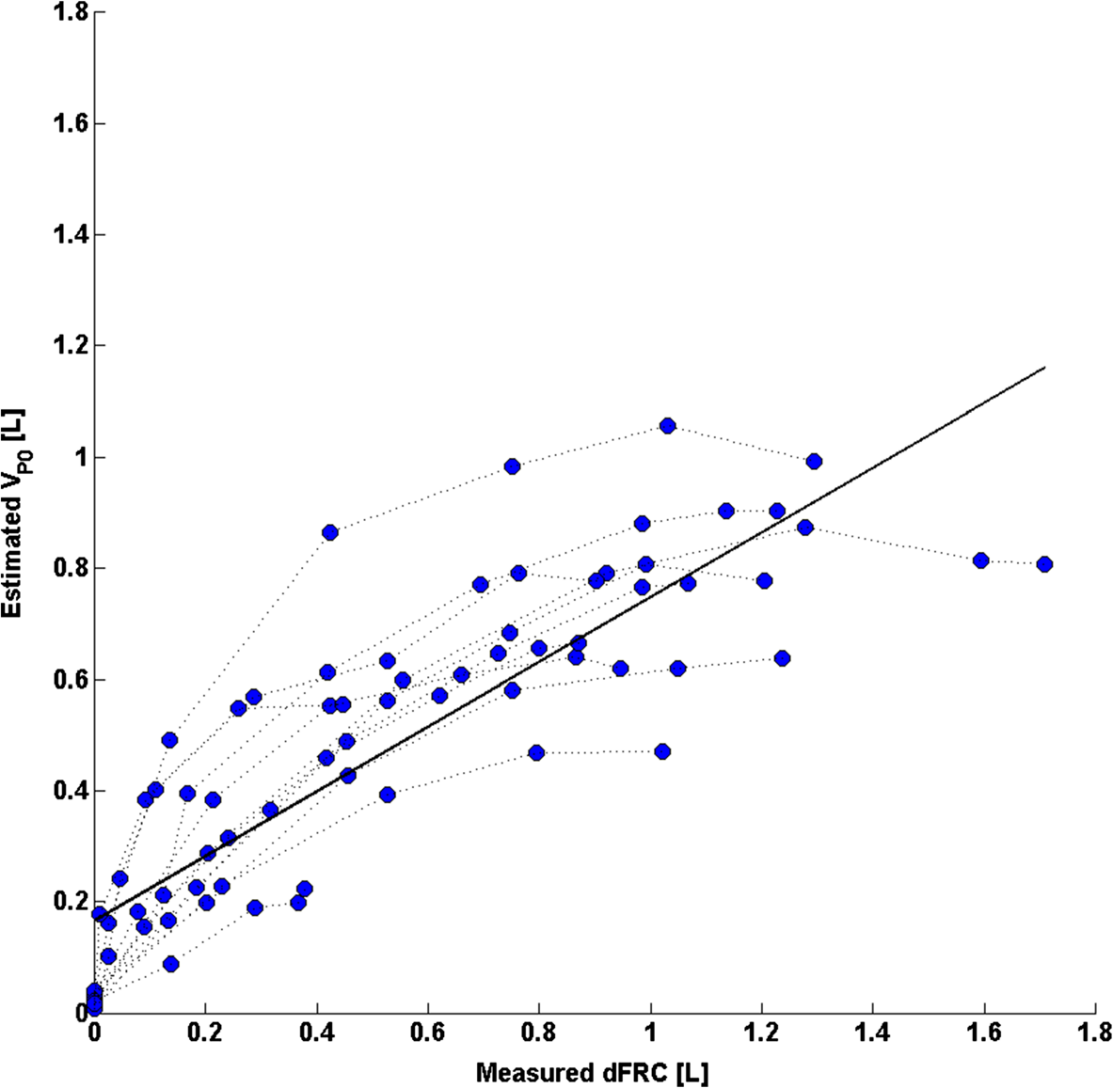
**Patient specific trends in cohort 1 between clinically measured dFRC and estimated*V***_***Po***_**for all patients and PEEP levels.** Patient specific trends are indicated to show general non-linearity.

The correlation coefficients observed in Figure 5 and Table 2 for the SCSB method indicate a possible linear relationship between measured dFRC and estimated *V*_*Po*_, indicating that *V*_*Po*_ may be linearly related to dFRC. This relationship is based on the assumption that the respiratory elastance, *E*_*rs*_, is the same in both Eqs. (4) and (15) which, if valid, would result in a strong correlation between these two elastance values for each patient. However, the scatter in Figure 8 indicates that this assumption may not be fully justified and may have resulted in estimation errors in the calculation of *V*_*Po*_. The error in *V*_*Po*_ across a range of PEEP levels can be seen in Figure 9 where the individual patient specific trends between measured dFRC and estimated *V*_*Po*_ from cohort 1 in Figure 5 have been highlighted. The relationship is seen to be non-linear with a concave response as PEEP (and consequently dFRC) increase. It is possible that some normalization of the data may correct for this.

### Combined method (CM)

The combined method incorporates the higher linear correlation observed with the SSMB method with the clinical applicability of the SSSB method. Because the combined method considers a progressive increase in the number of available PEEP levels, it can manage changes in compliance with less error than the SSMB method alone, which assumes a linear compliance trend over all PEEP levels, as well as versus the single breath methods. Overall, this approach is clinically feasible, practical and accurate for the range of clinically acceptable PEEP values seen in application.

### Outcomes

By using non-invasive model-based approaches, dFRC can be tracked continuously as it changes with the evolution of the disease. Although dFRC by itself only gives information on the additional lung volume due to PEEP changes, it can be used in conjunction with arterial blood gas measurements to help model the recruitment potential and response of the lung. In, particular, it might also be combined with existing models of gas exchange [25] to create a fully model-based approach to estimating lung recruitment.

The proposed methods have limitations in their predictive capability since in some cases the error observed between the measured and estimated values was exceptionally large. Auto-PEEP in particular has potential to affect the accuracy of the models. However, it can be detected directly from PV loop responses and thus managed. In the process of evaluating all four methods, it was found that the CM is the optimal method to estimate dFRC for real time application.

## Conclusions

The research presented evaluates four model-based methods for their capability of estimating dFRC for mechanically ventilated patients. By monitoring or tracking changes in patient respiratory mechanics, the clinician is able to evaluate the potential of recruitable lung in the patient. This may help to determine the optimal level of PEEP required during MV. In some cases, model limitations and large estimation errors limit the use of the methods for estimating recruitment potential. The models can be implemented in the ICU without the use of time-consuming methods such as CT scans.

## Abbreviations

ARDS: Acute Respiratory Distress Syndrome; ICU: Intensive Care Unit; MV: Mechanical ventilation; FRC: Functional Residual Capacity; PEEP: Positive End Expiratory Pressure; CT: Computed tomography; EIT: Electrical impedance tomography; dFRC: Dynamic Functional Residual Capacity; PV: Pressure-volume; SSMB: Stress-strain multiple breath; SSSB: Stress-strain single breath; SCSB: Single compartment single breath; CM: Combined method.

## Competing interests

There are no competing interests.

## Authors’ contribution

EJD developed the combined method, performed the validation of the models and drafted the manuscript. JGC participated in the implementation and coordination of the study and helped to draft the manuscript. YSC developed the single compartment single breath method and helped to draft the manuscript. GMS helped with the data acquisition. TD participated in the implementation and coordination of the study. All authors read and approved the final manuscript.

## Supplementary Material

Additional file 1 Median *β* [cmH_2_O] values for use in the SSMB method and the CM. Median *β*_*1*_ [cmH_2_O/mL] values for use in the SSSB method and the CM. Values of *β* and *β*_*1*_ determined for both data cohorts for use in the SSMB method, SSSB method and CM. Click here for file

Additional file 2 PEEP levels at which data was obtained for cohort 1 [24]. PEEP levels at which data was obtained for cohort 2 [9]. PEEP levels for cohorts 1 and 2 at which PV data was obtained. Click here for file
