# Supplementary material for: Analysis of different model-based approaches for estimating dFRC for real-time application
Source: Biomed Eng Online. 2013 Jan 31;12:9. doi: 10.1186/1475-925X-12-9 (PMC3599419; doi:10.1186/1475-925X-12-9)
Supplement: Additional file 1 — Median β [cmH2O] values for use in the SSMB method and the CM. Median β1 [cmH2O/mL] values for use in the SSSB method and the CM. Values of β and β1 determined for both data cohorts for use in the SSMB method, SSSB method and CM. [file 1475-925X-12-9-S1.docx]

Table 1 – Median *β* [cm H_2_O] values for use in SSMB method and CM.

| PEEP [cm H_2_O] | 0 | 5 | 7 | 10 | 12 | 15 | 20 | 25 | 30 |
| --- | --- | --- | --- | --- | --- | --- | --- | --- | --- |
| Cohort 1 [24] | -0.0026 | 3.0287 |  | 7.4455 |  | 12.6130 | 18.1691 | 23.8215 | 28.7313 |
| Cohort 2 [9] | 0.4825 | 4.4890 | 6.5108 | 9.3080 | 11.7909 | 13.7979 |  |  |  |

Table 2 – Median *β_1_* [cm H_2_O/mL] values for use in SSSB method and CM.

| PEEP [cm H_2_O] | 0 | 5 | 7 | 10 | 12 | 15 | 20 | 25 | 30 |
| --- | --- | --- | --- | --- | --- | --- | --- | --- | --- |
| Cohort 1 [24] | 0.0000 | 0.0081 |  | 0.0206 |  | 0.0348 | 0.0586 | 0.0802 | 0.0872 |
| Cohort 2 [9] | 0.0015 | 0.0140 | 0.0241 | 0.0362 | 0.0611 | 0.0796 |  |  |  |
