# Supplementary material for: Analysis of different model-based approaches for estimating dFRC for real-time application
Source: Biomed Eng Online. 2013 Jan 31;12:9. doi: 10.1186/1475-925X-12-9 (PMC3599419; doi:10.1186/1475-925X-12-9)
Supplement: Additional file 2 — PEEP levels at which data was obtained for cohort 1 [24]. PEEP levels at which data was obtained for cohort 2 [9]. PEEP levels for cohorts 1 and 2 at which PV data was obtained. [file 1475-925X-12-9-S2.docx]

Table 1 – PEEP levels at which data was obtained for cohort 1 [[24](#_ENREF_17)].

|  |  | PEEP [cm H_2_O] | | | | | | |
| --- | --- | --- | --- | --- | --- | --- | --- | --- |
|  |  | 0 | 5 | 10 | 15 | 20 | 25 | 30 |
| Trial | 1 | **●** | **●** | **●** | **●** | **●** | **●** |  |
|  | 2 | **●** | **●** | **●** | **●** | **●** |  |  |
|  | 3 | **●** | **●** | **●** | **●** | **●** | **●** |  |
|  | 4 | **●** | **●** | **●** | **●** | **●** | **●** | **●** |
|  | 5 | **●** | **●** | **●** | **●** | **●** | **●** |  |
|  | 6 | **●** | **●** | **●** | **●** | **●** | **●** |  |
|  | 7 | **●** | **●** | **●** | **●** | **●** | **●** |  |
|  | 8 | **●** | **●** | **●** | **●** | **●** |  |  |
|  | 9 | **●** | **●** | **●** | **●** |  |  |  |
|  | 10 | **●** | **●** | **●** | **●** | **●** | **●** | **●** |
|  | 11 | **●** | **●** | **●** | **●** | **●** | **●** | **●** |
|  | 12 | **●** | **●** | **●** | **●** | **●** | **●** |  |

Table 2 – PEEP levels at which data was obtained for cohort 2 [9].

|  | | PEEP [cm H_2_O] | | | | | |
| --- | --- | --- | --- | --- | --- | --- | --- |
|  | | 0 | 5 | 7 | 10 | 12 | 15 |
| Trial | 1 |  | **●** | **●** |  | **●** |  |
|  | 2 | **●** | **●** |  | **●** |  |  |
|  | 3 |  | **●** | **●** | **●** |  |  |
|  | 4 |  | **●** | **●** | **●** |  |  |
|  | 5 |  | **●** | **●** | **●** | **●** |  |
|  | 6 |  | **●** | **●** | **●** | **●** |  |
|  | 7 |  | **●** | **●** | **●** | **●** |  |
|  | 8 |  | **●** | **●** |  | **●** |  |
|  | 9 |  |  |  | **●** | **●** | **●** |
|  | 10 |  | **●** | **●** | **●** | **●** |  |
|  | 11 |  | **●** | **●** | **●** |  |  |
|  | 12 |  | **●** |  | **●** |  | **●** |
